# Regional perfusion and oxygenation of the kidney in an ovine model of severe sepsis with hypotension and kidney injury

**DOI:** 10.1186/cc11722

**Published:** 2012-11-14

**Authors:** P Calzavacca, K Ishikawa, K Lu, RG Evans, A Skene, R Bellomo, CN May

**Affiliations:** 1 Ospedale Uboldo, Cernusco sul Naviglio, Italy; 2 Iwate Medical University, Morioka, Japan; 3 Melbourne University, Florey Neuroscience Institutes, Parkville, Australia; 4 Monash University, Monash, Australia; 5 Austin Hospital, Heidelberg, Australia

## Background

The pathophysiology of septic acute kidney injury (AKI) is poorly understood. Renal medullary hypoxia has been proposed as a cause of AKI, but the changes in intrarenal oxygenation in hyperdynamic sepsis are unknown. Accordingly, we sought to determine the changes in regional renal perfusion and tissue oxygenation in the cortex and in the medulla in an ovine model of severe sepsis.

## Methods

Mean arterial pressure (MAP), cardiac index (CI) and renal blood flow (RBF) were continuously monitored in conscious sheep. Fibre optic probes (Oxford Optronix) were used to measure tissue perfusion and oxygen partial pressure at 30-second intervals. Arterial and renal venous blood samples were collected for oximetry. After 24 hour of baseline data collection, sepsis was induced with live *Escherichia coli *infusion for 24 hours. Gentamycin was given to terminate sepsis. The animals were followed for a further 24 hours of recovery. Histology was performed to confirm the position of the catheter tips and to assess tissue viability around the probes. Data are mean (± standard error) of the average of the periods. One-way repeated-measures ANOVA was used and *P *< 0.05 considered significant.

## Results

**Figure 1:**
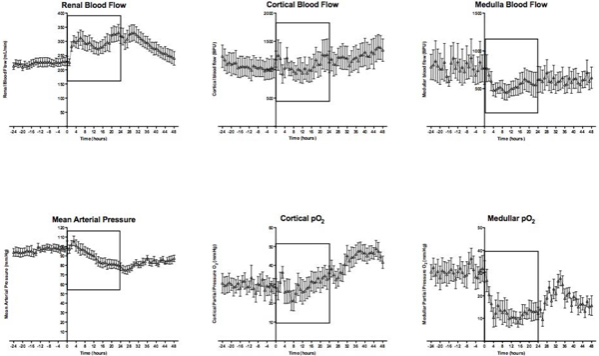
**Haemodynamic and regional perfusion/oxygenation changes**. Data are mean (standard error). Rectangle: 24-hour sepsis period.

Eight animals were studied. All animals developed a hyperdynamic state with a doubling in heart rate and CI, and a 50% increase in RBF. MAP decreased by 15 mmHg, with a fourfold increase in arterial lactate. Urine output halved, serum creatinine doubled and creatinine clearance decreased by one-third. Two animals died 28 hours after induction of sepsis. Baseline cortical and medullar pO_2 _were 29.6 (± 4.3) and 29.1 (± 4.1) mmHg, respectively. During sepsis (see Figure 1), cortical perfusion and oxygenation did not change significantly. In contrast, medullary perfusion showed an early trend towards reduction, while medullary tissue pO_2 _had decreased by 53 ± 11% at 24 hours of sepsis (*P *< 0.05). Calculated total renal oxygen consumption did not change significantly (from 33 to 32 ml O_2_/minute).

## Conclusion

This is the first study to measure intrarenal perfusion and oxygenation in hyperdynamic sepsis. In a conscious large animal model of septic AKI, we showed decreased medullary oxygenation, possibly due to mismatched local perfusion and oxygen consumption.

